# Supplementary material for: The integration of pharmacophore-based 3D QSAR modeling and virtual screening in safety profiling: A case study to identify antagonistic activities against adenosine receptor, A2A, using 1,897 known drugs
Source: PLoS One. 2019 Jan 3;14(1):e0204378. doi: 10.1371/journal.pone.0204378 (PMC6317804; doi:10.1371/journal.pone.0204378)

S1 Fig A-D. An enlarged version of Fig 2 A-D.

A

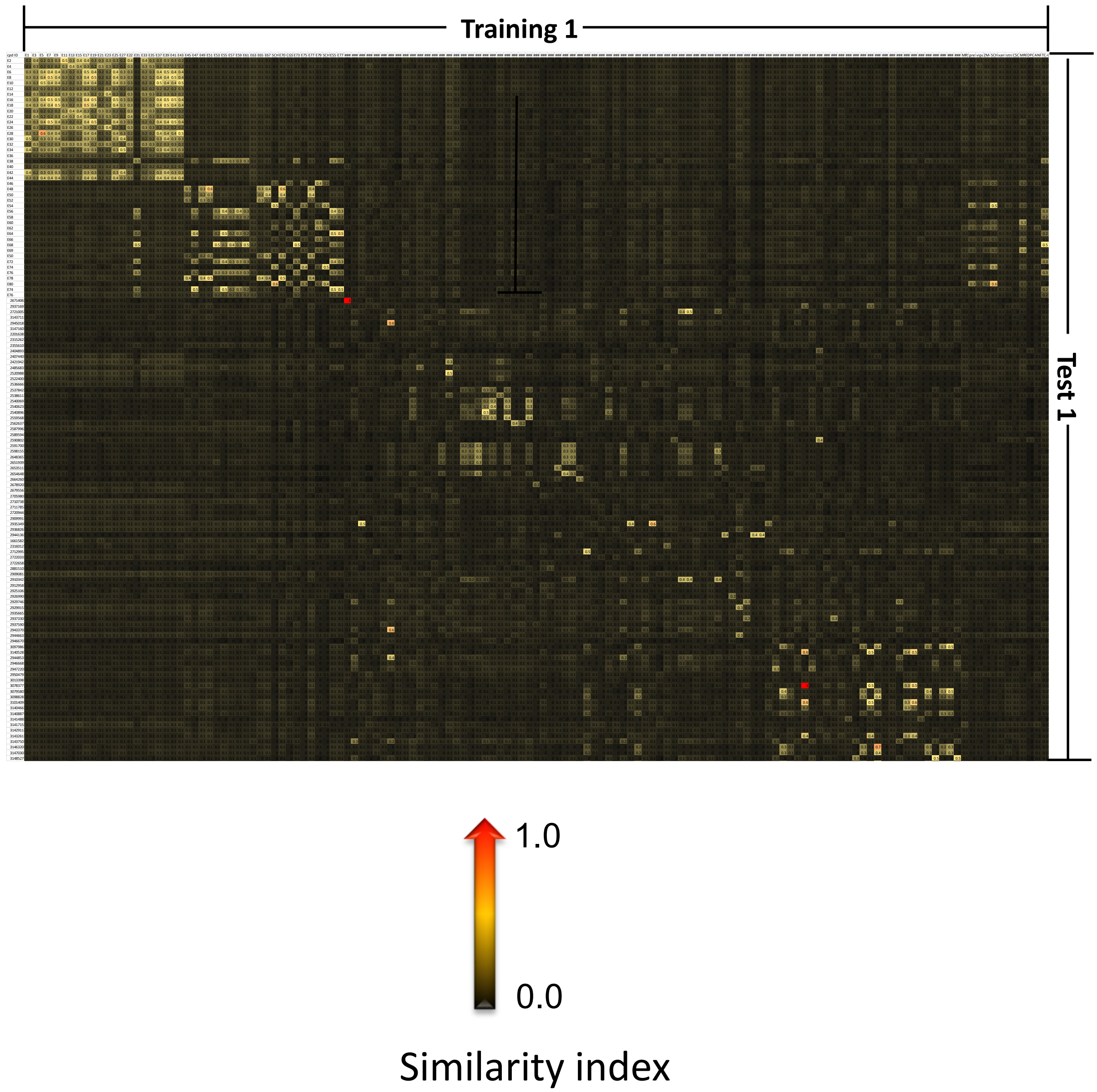

B

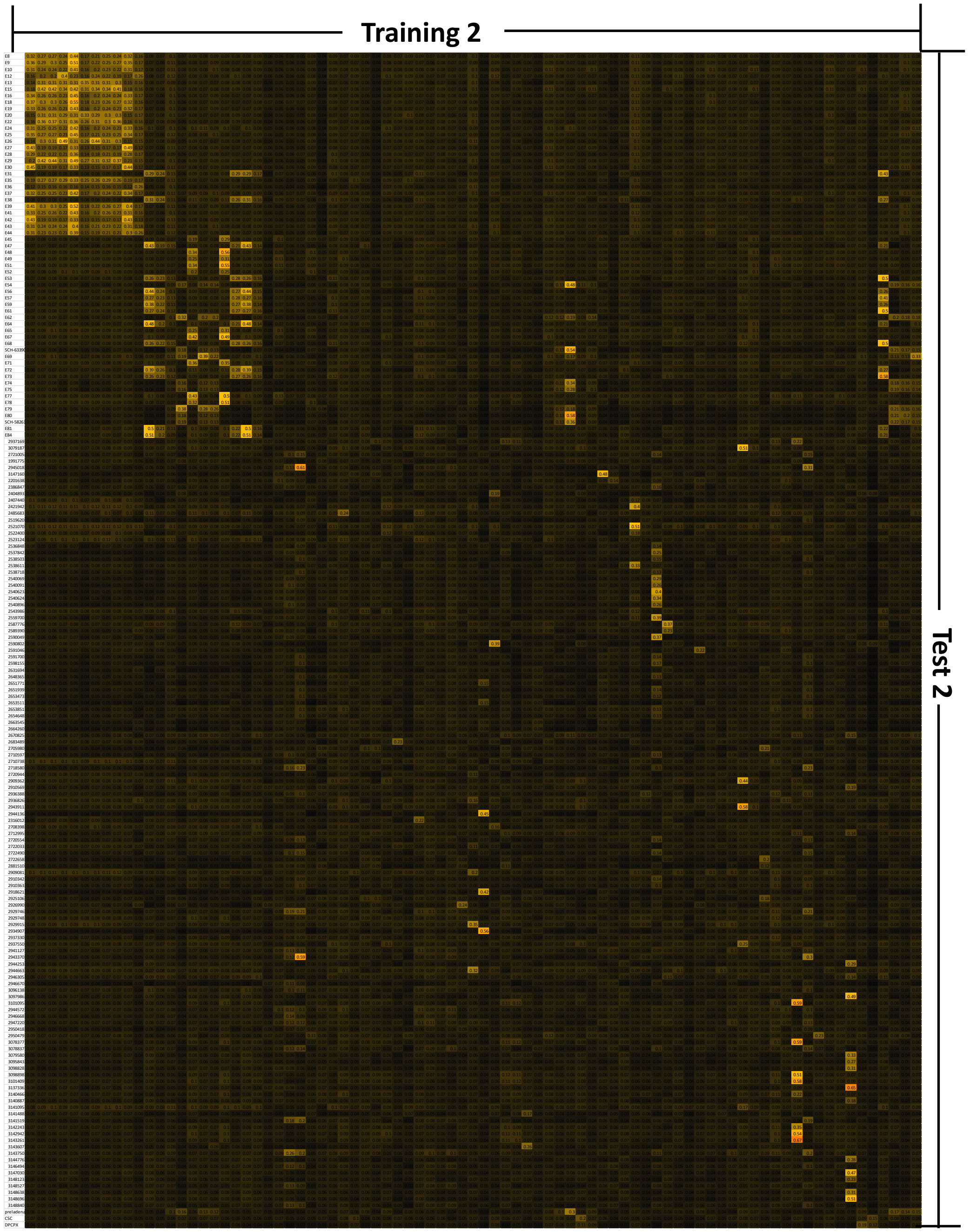

C

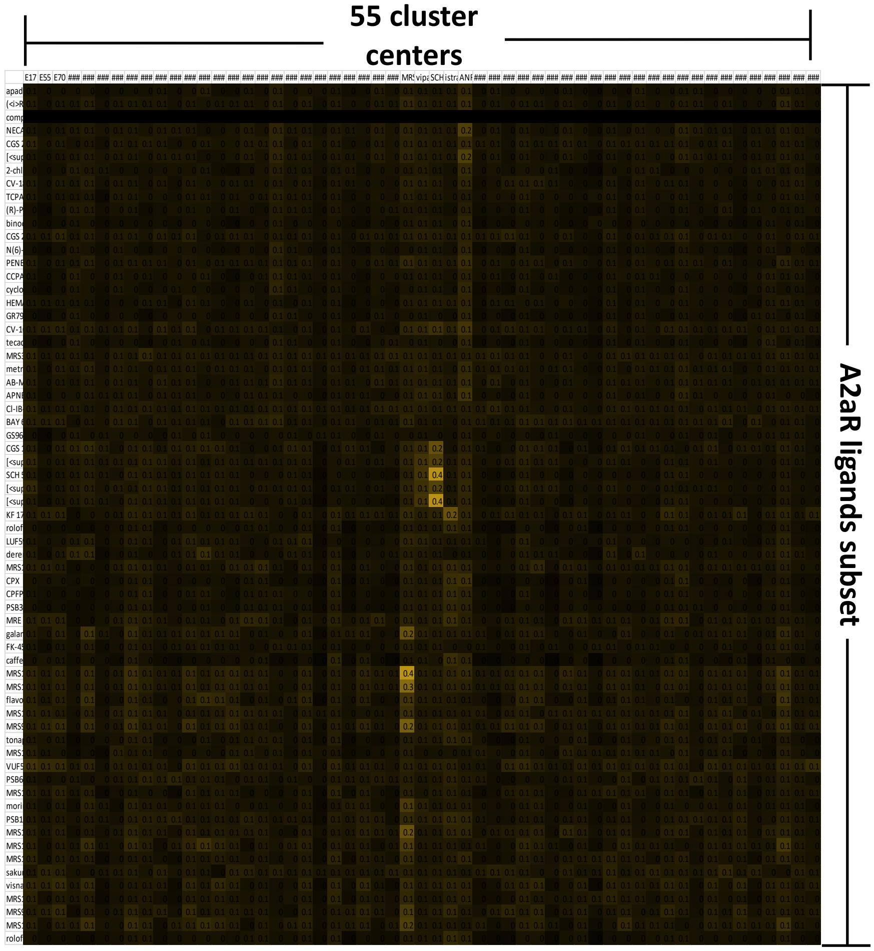

D

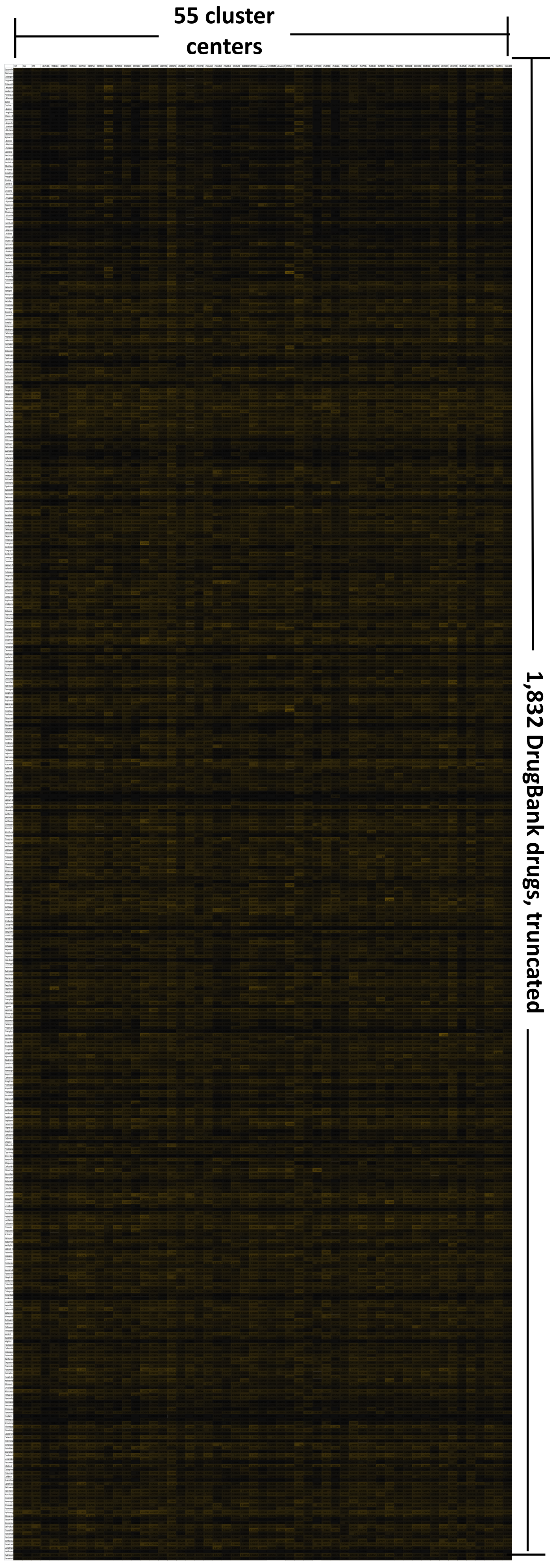

Supplement: S1 Fig — A-D. An enlarged version of Fig 2A–2D. (PDF) [file pone.0204378.s001.pdf]
