# Supplementary material for: The integration of pharmacophore-based 3D QSAR modeling and virtual screening in safety profiling: A case study to identify antagonistic activities against adenosine receptor, A2A, using 1,897 known drugs
Source: PLoS One. 2019 Jan 3;14(1):e0204378. doi: 10.1371/journal.pone.0204378 (PMC6317804; doi:10.1371/journal.pone.0204378)

S2 Fig. An example of QSAR visualization of positive and negative regression coefficients for active vs inactive molecules. **A**, pictorial representation of 5-methylthiophen-2-yl)(2-((pyrimidin-4-ylmethyl)amino)-thieno[3,2-d]pyrimidin-4-yl)methanone, an active whose measured pIC_{50} = 6.9. **B**, pictorial representation of 5-methoxy-2-((3-methyl-1-phenyl-4-(quinoxalin-6-yl)-1H-pyrazol-5-yl)amino)benzoic acid, an inactive whose measured pIC_{50} = 4.3.

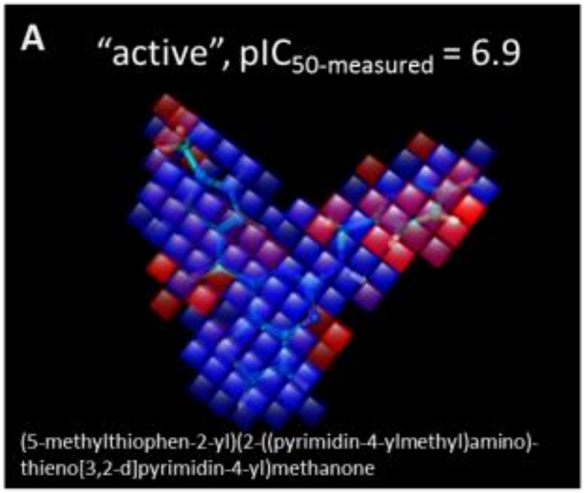

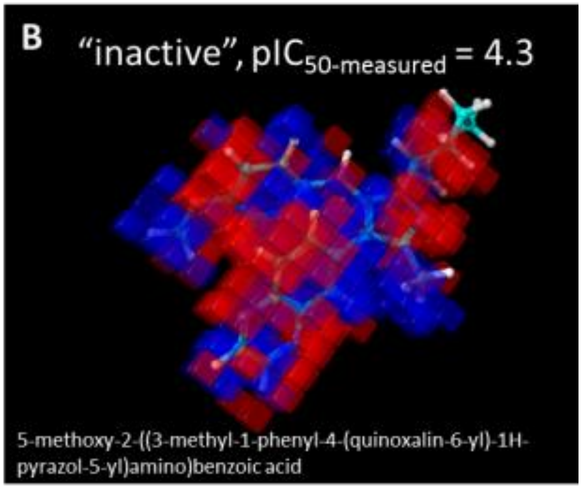

Supplement: S2 Fig — A-B. An example of QSAR visualization of positive and negative regression coefficients for active vs inactive molecule. (PDF) [file pone.0204378.s002.pdf]
