# Supplementary material for: The integration of pharmacophore-based 3D QSAR modeling and virtual screening in safety profiling: A case study to identify antagonistic activities against adenosine receptor, A2A, using 1,897 known drugs
Source: PLoS One. 2019 Jan 3;14(1):e0204378. doi: 10.1371/journal.pone.0204378 (PMC6317804; doi:10.1371/journal.pone.0204378)
Supplement: S1 Table — (PDF) [file pone.0204378.s003.pdf]

S1 Table. The list of 268 compounds used to build the QSAR model.

Compound	IC50, nM
Clc1cc2nc(NCc3cccnc3)nc(C(=O)c4cccs4)c2s1	62
Cc1ccc(s1)C(=O)c2nc(NCc3ccncn3)nc4ccsc24	116
Cc1ccc(s1)C(=O)c2nc(NCCc3cccnc3)nc4ccsc24	177
Cc1ccc(s1)C(=O)c2nc(N)nc3ccsc23	220
O=C(NC1CC1)c2nc(NCc3cccnc3)nc4ccsc24	710
Cc1oc(cc1)C(=O)c2nc(NCc3cccnc3)nc4ccsc24	1252
O=C(c1ccccc1)c2nc(NCc3cccnc3)nc4ccsc24	1619
Cc1ccc(cc1)C(=O)c2nc(NCc3cccnc3)nc4ccsc24	442
O=C(c1cncs1)c2nc(NCc3cccnc3)nc4ccsc24	1715
CCNC(=O)c1nc(NCc2cccnc2)nc3ccsc13	2516
Cc1ccc(s1)C(=O)c2nc(NCc3ccccn3)nc4ccsc24	186
CC(Nc1nc(C(=O)N2CCCC2)c3sccc3n1)c4cccnc4	2347
Cc1ccc(s1)C(=O)c2nc(C)nc3ccsc23	7847
CC(Nc1nc(C(=O)c2ccc(C)s2)c3sccc3n1)c4cccnc4	99
CCNc1nc(C(=O)c2ccc(C)s2)c3sccc3n1	126
O=C(c1ccsc1)c2nc(NCc3cccnc3)nc4ccsc24	405
Cc1ccc(s1)C(=O)c2nc(NCc3cccnc3)nc4ccsc24	990
Clc1ccc(s1)C(=O)c2nc(NCc3cccnc3)nc4ccsc24	1541
O=C(c1ccncc1)c2nc(NCc3cccnc3)nc4ccsc24	1942
CCc1nc(C(=O)c2ccc(C)s2)c3sccc3n1	3852
Cc1ccc(s1)C(=O)c2ncnc3ccsc23	6685
Cc1ccc(s1)C(=O)c2nc(NCC3CCCCC3)nc4ccsc24	10000
CC(Nc1nc(C(=O)c2ccc(C)s2)c3sccc3n1)c4ccccn4	118
COc1ccc(cc1)C(=O)c2nc(NCc3cccnc3)nc4ccsc24	637
O=C(c1nccs1)c2nc(NCc3cccnc3)nc4ccsc24	732
CC(Nc1nc(C(=O)c2ccc(C)s2)c3sccc3n1)c4ccccc4	1077
CCNc1cc2nc(NCc3cccnc3)nc(C(=O)c4cccs4)c2s1	2485
O=C(NC1CCCCC1)c2nc(NCc3cccnc3)nc4ccsc24	3093
Cc1ccc(s1)C(=O)c2nc(NCc3ccccc3)nc4ccsc24	7146
Nc1cc2nc(NCc3cccnc3)nc(C(=O)c4cccs4)c2s1	10000
CN(C)c1cccc(n1)c2cc(NC(=O)C)nc(n2)n3nc(C)cc3C	156
Cc1ccc(s1)C(=O)c2nc(NC(=O)c3cccnc3)nc4ccsc24	161
Cc1ccc(s1)C(=O)c2nc(NCCc3ccccn3)nc4ccsc24	351
CNc1cc2nc(NCc3cccnc3)nc(C(=O)c4cccs4)c2s1	965
CN(Cc1cccnc1)c2nc(C(=O)c3ccc(C)s3)c4sccc4n2	2271
C[C@@H](Nc1nc(C(=O)N2CCCC2)c3sccc3n1)c4cccnc4	6760
CNC(=O)c1nc(NCc2cccnc2)nc3ccsc13	7107
COc1cccc(c1F)c2cc(NC(=O)C)nc(n2)n3nc(C)cc3C	32
O=C(c1cccs1)c2nc(NCc3cccnc3)nc4ccsc24	51
C[C@H](Nc1nc(C(=O)N2CCCC2)c3sccc3n1)c4cccnc4	468
O=C(c1cccnc1)c2nc(NCc3cccnc3)nc4ccsc24	1168

CN(C)c1cc2nc(NCc3cccnc3)nc(C(=O)c4cccs4)c2s1	1417
CC(C)NC(=O)c1nc(NCc2cccnc2)nc3ccsc13	2610
O=C(N1CCCC1)c2nc(NCc3cccnc3)nc4ccsc24	6136
Cc1ccccc1n2nc(C)c(c3cc(F)c4nccnc4c3)c2Nc5ccccc5C(=O)O	50000
CCCSc1c2c(nc(N)n3nc(nc23)c4occc4)nn1C	14.8
COc1cncc(c1)c2cc(NC(=O)CN3CCOCC3)nc(n2)n4nc(C)cc4C	49
Cc1ccc(Nc2c(c(C)nn2c3ccccc3C)c4ccc5nccnc5c4)c(c1)C(=O)O	50000
Cc1ccccc1n2nc(c(c3ccc4nccnc4c3)c2Nc5ccccc5C(=O)O)C(F)(F)F	50000
COc1ccc(Nc2c(c(C)nn2c3ccccc3)c4ccc5nccnc5c4)c(c1)C(=O)O	5500
Cc1ccccc1n2nc(C)c(c3ccc4nccnc4c3)c2Nc5ccc(Cl)cc5C(=O)O	50000
Cc1ccccc1n2ncc(c3ccc4nccnc4c3)c2Nc5ccccc5C(=O)O	50000
CC(=O)Nc1cc(nc(n1)n2nc(C)cc2C)c3cccc(n3)N4CCOCC4	13
COc1ccc(CCCn2ncc3c2nc(N)n4nc(nc34)c5occc5)cc1OC	20
COc1cncc(c1)c2cc(NC(=O)C)nc(n2)n3nc(C)cc3C	57
CC(=O)Nc1cc(nc(n1)n2nc(C)cc2C)c3cncc(O)c3	64
COCCN(C)c1cccc(n1)c2cc(NC(=O)C)nc(n2)n3nc(C)cc3C	70
COC[C@H]1CCCN1c2cc(NC(=O)C)nc(n2)n3nc(C)cc3C	85
CC(=O)Nc1cc(nc(n1)n2nc(C)cc2C)c3cncc(c3)N4CCOCC4	213
COc1ccc(NC(=O)Nc2nc3nn(C)c(SC)c3c4nc(nn24)c5occc5)cc1	17.5
COC1CN(C1)c2cccc(n2)c3cc(NC(=O)C)nc(n3)n4nc(C)cc4C	18
Cl.CCNc1c2c(nc(N)n3nc(nc23)c4occc4)nn1C	48
CN1CCN(CC(=O)Nc2cc(nc(n2)c3occc3)c4nccs4)CC1	103
CCOC(=O)Nc1cc(nc(n1)n2nc(C)cc2C)c3cncc(OC)c3	196
CCc1nn(c(Nc2ccccc2C(=O)O)c1c3ccc4nccnc4c3)c5ccccc5C	50000
CSc1c2c(nc(NC(=O)Cc3ccc4OCOc4c3)n5nc(nc25)c6occc6)nn1C	21.5
CCc1ccccc1n2nc(C)c(c3ccc4nccnc4c3)c2Nc5ccc(OC)cc5C(=O)O	50000
CN1CCN(CC1)c2cccc(n2)c3cc(NC(=O)C)nc(n3)n4nc(C)cc4C	7
Nc1nc2c(cnn2CCCc3ccccc3)c4nc(nn14)c5occc5	18
Cl.CCNc1c2c(nc(NC(=O)Nc3ccc(OC)cc3)n4nc(nc24)c5occc5)nn1C	61
COc1ccc(Nc2c(c(C)nn2c3ccccc3C)c4ccc5nccnc5c4)c(c1)C(=O)O	50000
COc1ccc(Nc2c(c(C)nn2C3CCCCC3)c4ccc5nccnc5c4)c(c1)C(=O)O	50000
Nc1nc2ccc(Cl)cc2c3nc(nn13)c4occc4	12
COc1cc(OC)cc(c1)c2cc(NC(=O)C)nc(n2)n3nc(C)cc3C	17
CC(=O)Nc1cc(nc(n1)n2nc(C)cc2C)c3cccc(n3)N4CCC(O)CC4	22
Nc1nc2c(cnn2CCc3ccc(OCc4ccccc4)cc3)c5nc(nn15)c6occc6	90
Nc1nc2c(cnn2Cc3ccc(OCc4ccccc4)cc3)c5nc(nn15)c6occc6	500
CC(=O)Nc1cc(nc(n1)n2nc(C)cc2C)N3CCCC3=O	1000
COc1ccc(Nc2c(c(C)nn2c3ccccc3Cl)c4ccc5nccnc5c4)c(c1)C(=O)O	50000
Cc1ccccc1n2nc(C)c(c3ccc4nccnc4c3)c2Nc5ccccc5C(=O)O	50000
CSc1c2c(nc(N)n3nc(nc23)c4occc4)nn1C	6.1
Nc1nc2c(cnn2CCCc3ccc(O)cc3)c4nc(nn14)c5occc5	10
Nc1nc2c(cnn2CCc3ccccc3)c4nc(nn14)c5occc5	12
CCC(=O)Nc1cc(nc(n1)n2nc(C)cc2C)c3cncc(OC)c3	31
COc1cncc(c1)c2cc(NC(=O)C)nc(n2)n3nc(C)cc3C	85

Cc1cc(C)n(n1)c2cc(NC(=O)Cc3ccc(cc3)S(=O)(=O)C)nc(n2)c4ccccn4	100
COC(=O)Nc1cc(nc(n1)n2nc(C)cc2C)c3cncc(OC)c3	167
AMG2671406	97.5
AMG2944978	229
AMG2937169	11500
AMG3079187	12000
AMG2721005	13600
AMG1900463	18300
AMG3143711	19100
AMG1991775	2280.9
AMG2945018	2690
AMG2539851	6096.8
AMG3147160	6810
AMG2938386	7590
AMG2201638	N/A
AMG2206979	N/A
AMG2315262	N/A
AMG2339263	N/A
AMG2355610	N/A
AMG2386847	N/A
AMG2404893	N/A
AMG2407410	N/A
AMG2407440	N/A
AMG2409753	N/A
AMG2421942	N/A
AMG2422813	N/A
AMG2485683	N/A
AMG2519620	N/A
AMG2520988	N/A
AMG2521070	N/A
AMG2522400	N/A
AMG2523124	N/A
AMG2536666	N/A
AMG2536848	N/A
AMG2537842	N/A
AMG2538503	N/A
AMG2538611	N/A
AMG2538718	N/A
AMG2540069	N/A
AMG2540091	N/A
AMG2540623	N/A
AMG2540624	N/A
AMG2540896	N/A
AMG2543986	N/A

AMG2559568	N/A
AMG2559700	N/A
AMG2562637	N/A
AMG2587776	N/A
AMG2587996	N/A
AMG2589390	N/A
AMG2589594	N/A
AMG2590049	N/A
AMG2590802	N/A
AMG2591046	N/A
AMG2591700	N/A
AMG2591846	N/A
AMG2598155	N/A
AMG2631694	N/A
AMG2648365	N/A
AMG2651771	N/A
AMG2651939	N/A
AMG2653473	N/A
AMG2653511	N/A
AMG2653851	N/A
AMG2654648	N/A
AMG2663545	N/A
AMG2664260	N/A
AMG2670825	N/A
AMG2678920	N/A
AMG2679213	N/A
AMG2679556	N/A
AMG2683489	N/A
AMG2705980	N/A
AMG2710597	N/A
AMG2710738	N/A
AMG2710917	N/A
AMG2711785	N/A
AMG2718580	N/A
AMG2720944	N/A
AMG2909362	N/A
AMG2909991	N/A
AMG2910569	N/A
AMG2935349	N/A
AMG2936388	N/A
AMG2936826	N/A
AMG2943911	N/A
AMG2944136	N/A
AMG1077285	N/A

AMG1661582	N/A
AMG2204440	N/A
AMG2316012	N/A
AMG2708398	N/A
AMG2712995	N/A
AMG2720554	N/A
AMG2722033	N/A
AMG2722490	N/A
AMG2722658	N/A
AMG2723901	N/A
AMG2881510	N/A
AMG2885592	N/A
AMG2909081	N/A
AMG2909256	N/A
AMG2910342	N/A
AMG2910363	N/A
AMG2912958	N/A
AMG2918621	N/A
AMG2925106	N/A
AMG2926829	N/A
AMG2926990	N/A
AMG2929672	N/A
AMG2929746	N/A
AMG2929748	N/A
AMG2929915	N/A
AMG2934907	N/A
AMG2935665	N/A
AMG2937250	N/A
AMG2937330	N/A
AMG2937550	N/A
AMG2937590	N/A
AMG2941127	N/A
AMG2943370	N/A
AMG2944253	N/A
AMG2944663	N/A
AMG2946305	N/A
AMG2946670	N/A
AMG3096138	N/A
AMG3097986	N/A
AMG3101095	N/A
AMG3140528	N/A
AMG2944572	N/A
AMG2944853	N/A
AMG2946460	N/A

AMG2946668	N/A
AMG2946850	N/A
AMG2947220	N/A
AMG2950418	N/A
AMG2950479	N/A
AMG2950853	N/A
AMG3013398	N/A
AMG3013528	N/A
AMG3078377	N/A
AMG3078837	N/A
AMG3079580	N/A
AMG3095843	N/A
AMG3098828	N/A
AMG3098898	N/A
AMG3101409	N/A
AMG3137336	N/A
AMG3140466	N/A
AMG3140883	N/A
AMG3140887	N/A
AMG3141095	N/A
AMG3141488	N/A
AMG3141519	N/A
AMG3141715	N/A
AMG3142243	N/A
AMG3142911	N/A
AMG3142942	N/A
AMG3143261	N/A
AMG3143607	N/A
AMG3143750	N/A
AMG3144776	N/A
AMG3146320	N/A
AMG3146494	N/A
AMG3147030	N/A
AMG3148123	N/A
AMG3148527	N/A
AMG3148638	N/A
AMG3148696	N/A
AMG3148840	N/A
CCOc1cc(O)c2c(c1)oc(c(c2=O)OCC)c1ccccc1	0.630957
COCCOc1ccc(cc1)N1CCN(CC1)CCn1ncc2c1nc(N)n1c2nc(n1)c1ccco1	0.794328
Nc1nc(c2ccco2)c2c(n1)n(nn2)Cc1ccc(c(c1)C)N	1.258925
Oc1ccc(cc1)CCNc1nc(N)n2c(n1)nc(n2)c1ccco1	1.258925
COc1ccc(cc1)CCCn1ncc2c1nc(N)n1c2nc(n1)c1ccco1	1
CCCn1c2nc([nH]c2c(=O)n(c1=O)CCC)c1ccc(cc1)OCC(=O)NCCN	1.995262

COc1cc(C=Cc2nc3c(n2C)c(=O)n(c(=O)n3CC)CC)ccc1OC	3.162278
Clc1cccc(c1)C=Cc1nc2c(n1C)c(=O)n(c(=O)n2C)C	50.11872
CCCn1cc2c(n1)nc(n1c2nc(n1)c1ccco1)NC(=O)Nc1ccc(cc1)OC	158.4893
CCCn1c2nc([nH]c2c(=O)n(c1=O)CCC)C1CCCC1	125.8925
NC1=C2N=C(OCC)N(CC)C2=NC=N1	50.11872
CC(NC1=NC(N2N=C(C)C=C2C)=NC(C3=NC(N4CCC(OC)CC4)=CC=C3)=C1)=O	0.398107
Cn1c(=O)n(C)c2c(c1=O)[nH]cn2	3162.278
